# Deleted in Breast Cancer 1 regulates cellular senescence during obesity

**DOI:** 10.1111/acel.12235

**Published:** 2014-07-03

**Authors:** Carlos Escande, Veronica Nin, Tamar Pirtskhalava, Claudia C Chini, Maria Thereza Barbosa, Angela Mathison, Raul Urrutia, Tamar Tchkonia, James L Kirkland, Eduardo N Chini

**Affiliations:** 1 Department of Anesthesia, Mayo ClinicRochester, MN, USA; 2 Robert and Arlene Kogod Center on Aging, Mayo ClinicRochester, MN, USA; 3 Institut Pasteur MontevideoMontevideo, Uruguay; 4 Laboratory of Epigenetics and Chromatin Dynamics, Mayo ClinicRochester, MN, USA; 5 Epigenomic Translational Program, Mayo Clinic Center for Individualized MedicineRochester, MN, USA

**Keywords:** aging, hdacs, mice, obesity, senescence, signaling, Sir2

## Abstract

Chronic obesity leads to inflammation, tissue dysfunction, and cellular senescence. It was proposed that cellular senescence during obesity and aging drives inflammation and dysfunction. Consistent with this, clearance of senescent cells increases healthspan in progeroid mice. Here, we show that the protein Deleted in Breast Cancer-1 (DBC1) regulates cellular senescence during obesity. Deletion of DBC1 protects preadipocytes against cellular senescence and senescence-driven inflammation. Furthermore, we show protection against cellular senescence in DBC1 KO mice during obesity. Finally, we found that DBC1 participates in the onset of cellular senescence in response to cell damage by mechanism that involves binding and inhibition of HDAC3. We propose that by regulating HDAC3 activity during cellular damage, DBC1 participates in the fate decision that leads to the establishment of cellular senescence and consequently to inflammation and tissue dysfunction during obesity.

## Introduction

**Figure 1:**
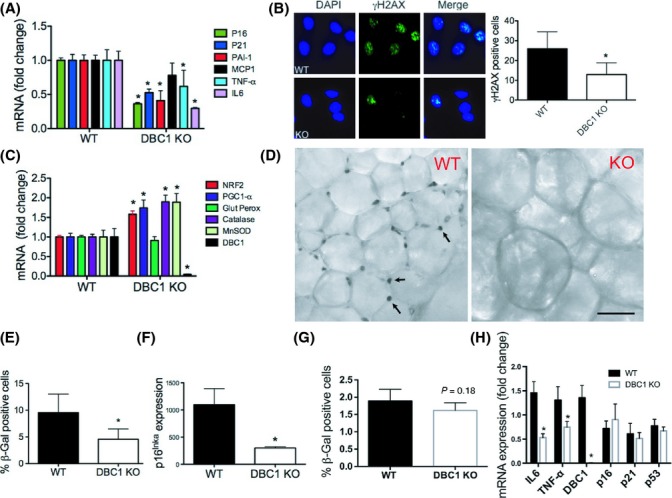
Deletion of DBC1 protects against cellular senescence during obesity (A) Cellular senescence and SASP marker gene expression by RT–PCR in cultured inguinal mouse preadipocytes after HFD. (B) Left, γ-H2.AX immunostaining in preadipocytes from WT and DBC1 KO mice. Right, quantification of γ-H2.AX foci-positive cells (*P < 0.05; *t*-test; *n* = 4 mice/group). (C) ROS and mitochondrial function marker gene expression by RT–PCR in cultured preadipocytes (*P < 0.05; *t*-test; *n* = 4 mice/ group). (D) SA-βGal activity in inguinal adipose tissue of WT and DBC1 KO mice after 12 weeks on a high-fat diet. Arrowheads point to positive cells. (E) Quantitation of cellular SA-βGal activities in the inguinal fat of the mice described in D (*P* < 0.05; *t*-test; *n* = 4 mice/ group). (F) Expression of the senescence marker, p16^INK^^4a^, by RT–PCR in inguinal fat under the conditions described in D. (G–H) Senescence and inflammation markers in inguinal fat tissue of WT and DBC1 KO female mice at 16 months of age fed with normal chow diet. (G) Quantitation of SA-βGal activity. H) Expression of senescence and inflammation markers by RT–PCR. (*P* < 0.05; t-test; *n* = 4 mice/ group)

Obesity, a major health problem in the USA and many developed countries (Flegal *et al*., 2012), is associated with an increase in cellular senescence and inflammation (Tchkonia *et al*., 2010). Cellular senescence has been proposed to promote chronic, “sterile” inflammation through the senescence-associated secretory phenotype (SASP) (Tchkonia *et al*., 2010). Supporting this notion, some of us found that eliminating senescent cells from progeroid mice improves healthspan (Baker *et al*., 2011). The physiological and molecular events that lead to cellular senescence, however, are still poorly understood. We have been studying the role of the protein Deleted in Breast Cancer-1 (DBC1) in energy metabolism (Chini *et al*., 2013). DBC1 regulates several nuclear proteins, including SIRT1 and HDAC3 (Escande *et al*., 2010; Chini *et al*., 2013). Both SIRT1 and HDAC3 regulate cellular senescence (Ghosh, 2008; Feng *et al*., 2009). We investigated whether DBC1 plays a role in cellular senescence and the SASP during obesity. We found that preadipocytes isolated from WT and DBC1 KO mice after 12 weeks of high-fat diet feeding exhibit less senescence, indicated by lower levels of p16^Ink4a^ and p21, as well as the SASP markers, MCP-1, TNF-α, and IL-6 (Fig. 1A). Also, we found fewer γ-H2.AX (a marker of activated DNA damage responses)-positive preadipocytes isolated from DBC1 KO mice (Fig. 1B). Several markers of antioxidant defense mechanisms were up-regulated in preadipocytes from DBC1 KO mice (Fig. 1C). Consistent with our in vitro results, DBC1 KO mice have less cellular senescence in adipose tissue during high-fat diet feeding measured by cytoplasmic (Fig. S1A) SA-βGal activity and p16^Ink4a^ expression (Fig. 1D–F). The effect of DBC1 on cellular senescence may not be linked to chronological aging, as there was no difference between WT and DBC1 KO mice fed with normal chow during 16 months (Fig. 1G–H). Nevertheless, DBC1 KO mice had less inflammation in fat tissue (Fig. 1H). We are currently investigating whether there is a difference on cellular senescence that may appear later in life.

**Figure 2:**
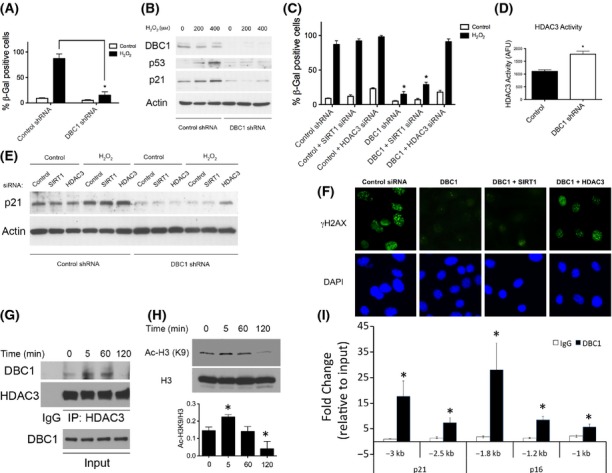
DBC1 regulates cellular senescence by an HDAC3-mediated mechanism (A) Quantification of cellular SA-βGal activity in 3T3-L1 preadipocytes following H_2_O_2_ treatment (200 μm; *P < 0.05; *t*-test; *n* = 5). (B) Protein expression of p53 and p21 in 3T3-L1 preadipocytes stably transfected with scrambled or DBC1 shRNA and treated with 200 μm H_2_O_2_. (C) Quantitation of SA-βGal staining in 3T3-L1 after treatment with H_2_O_2_ (200 μm). Cells stably transfected with control or DBC1 shRNA were transfected with control, SIRT1, or HDAC3 siRNA before H_2_O_2_ treatment. Senescence was evaluated by cellular SA-βGal activity (*P < 0.05; t-test; *n* = 5). (D) HDAC3 deacetylase activity measured after immunoprecipitation of HDAC3 preadipocytes stably transfected with control or DBC1 shRNA. (E) Representative effect of SIRT1 and HDAC3 knockdown on the effect of DBC1 in p21 expression after H_2_O_2_ treatment. (*n* = 3) (F) Effect of DBC1, SIRT1, and HDAC3 siRNA on γ-H2.AX foci in 3T3-L1 preadipocytes after incubation with H_2_O_2_ (200 μm) (*n* = 3). (G) Time-dependent interaction between HDAC3 and DBC1 after treatment of 3T3-L1 preadipocytes with 200 μm of H_2_O_2_. (H) Upper, time dependence of histone H3 lysine residue 9 acetylation (K9) after treatment of preadipocytes with 200 μm H_2_O_2_. Lower, densitometry analysis of K9 histone 3 acetylation (*P < 0.05; *t*-test; *n* = 3). (I) Chromatin immunoprecipitation (ChIP) for the p21 and p16 promoter regions in 3T3-L1 preadipocytes using an antibody against DBC1. Nonspecific IgG was used as control. The results shown are the average ± SEM of 4 independent ChIP. (*P < 0.01; *t*-test)

Next, we investigated whether deletion of DBC1 protects against DNA damage-induced cellular senescence. We induced DNA damage by H_2_O_2_ treatment in 3T3-L1 preadipocytes stable expressing scrambled shRNA (Control shRNA) or DBC1 shRNA. We found increased cellular SA-βGal activity in the control shRNA cells exposed to H_2_O_2_, but not in cells expressing DBC1 shRNA (Fig. 2A). Control cells showed a dose-dependent increase in expression of p53 and p21 after H_2_O_2_ treatment. However, there were no changes in p53 and p21 in cells expressing DBC1 shRNA (Fig. 2A). The effect of DBC1 on the response to H_2_O_2_-induced DNA damage was only related to cellular senescence, as apoptosis was not affected by DBC1 knockdown (Fig. S1B). DBC1 binds and inhibits HDAC3 (Chini *et al*., 2010). Indeed, HDAC3 regulates DNA damage response (Bhaskara *et al*., 2010) and inhibits expression of the senescence mediator p16^Ink4a^ (Zheng *et al*., 2012). We found that the effect of DBC1 knockdown on senescence was completely abrogated by cotransfection with HDAC3 siRNA, but not by SIRT1 siRNA (Fig. 2C and Fig. S1C). Indeed, DBC1 knockdown increased HDAC3 activity in 3T3-L1 cells (Fig. 2D). Furthermore, HDAC3 siRNA, restored p21 expression driven by H_2_O_2_ treatment in DBC1 shRNA-expressing cells (Fig. 2E and Fig. S1D). Also, knockdown of DBC1 resulted in less γ-H2.AX-positive cells (Fig. 2F and Fig. S1E), an effect that was lost when HDAC3 was knocked down together with DBC1 (Fig. 2F). Interestingly, treatment with H_2_O_2_ led to a rapid increase in DBC1 binding to HDAC3 (Fig. 2G), which correlated with an increase in histone H3 acetylation (Ac-H3K9, Fig. 2H), a target site for HDAC3 (Bhaskara *et al*., 2010). Finally, we found that DBC1 is present in both p16 and p21 promoter regions in 3T3-L1 cells (Fig. 2I), with a binding profile similar to the one of HDAC3 (Fig. S1F), which suggests that DBC1 binding to the chromatin is bridged by HDAC3. DBC1 is regulated by the checkpoint kinase ATM (Yuan *et al*., 2012), and HDAC3 is required for the DNA damage response (Bhaskara *et al*., 2010). We propose that during the cellular stress driven by obesity, DBC1 has an active role in the onset of cellular senescence and inflammation. It is plausible that in the event of chronic damage or stress, DBC1 plays a role in checkpoint control, contributing to a switch in cell fate and promoting cellular senescence.

## Supporting Information

**Fig. S1** (A) DAPI counterstaining of fat tissue SA-βGal staining described in Figure 1D, showing cytoplasmic localization of the βGal signal. (B) Effect of DBC1 knockdown on apoptosis triggered by H_2_O_2_ in 3t3-L1 preadipocytes. Cells were incubated with 200 μm H_2_O_2_ for 2 h, washed and let them recover for 4 more hours. Apoptosis was determined by nuclear shape using DAPI as nuclear marker. Pictures were taken blindly before and after treatment and apoptosis was independently evaluated by counting cells in the field based in nuclear shape, size, and DNA condensation. Results shown represent average ± SEM of 3 independent experiments. (C) Western blot for DBC1, HDAC3, and SIRT1 in H_2_O_2_–treated 3T3-L1 preadipocytes transfected with the different siRNAs and collected at the time of H_2_O_2_ treatment. (D) Densitometry analysis for p21 expression in three independent experiments corresponding to the results shown in Figure 2E. (E) Quantitation of the effect of DBC1, SIRT1, and HDAC3 siRNA on γ-H2.AX foci in 3T3-L1 preadipocytes after incubation with H_2_O_2_ (200 μm) shown in Figure 2F. Connecting lines show significant differences between conditions (*P* < 0.05, ANOVA, *n* = 3). (F) Chromatin immunoprecipitation (ChIP) for the p21 and p16 promoter regions in 3T3-L1 preadipocytes using an antibody against HDAC3. Nonspecific IgG was used as control. The results shown are the average ± SEM of 4 independent ChIP. (**P* < 0.01; *t*-test). **Data. S1** Methods.
