# Supplementary material for: Deleted in Breast Cancer 1 regulates cellular senescence during obesity
Source: Aging Cell. 2014 Jul 3;13(5):951–3. doi: 10.1111/acel.12235 (PMC4172532; doi:10.1111/acel.12235)
Supplement: Supplementary file 1 — Fig. S1 (A) DAPI counterstaining of fat tissue SA-βGal staining described in Figure 1D, showing cytoplasmic localization of the βGal signal. (B) Effect of DBC1 knockdown on apoptosis triggered by H2O2 in 3t3-L1 preadipocytes. Cells were incubated with 200 μm H2O2 for 2 h, washed and let them recover for 4 more hours. Apoptosis was determined by nuclear shape using DAPI as nuclear marker. Pictures were taken blindly before and after treatment and apoptosis was independently evaluated by counting cells in the field based in nuclear shape, size, and DNA condensation. Results shown represent average ± SEM of 3 independent experiments. (C) Western blot for DBC1, HDAC3, and SIRT1 in H2O2–treated 3T3-L1 preadipocytes transfected with the different siRNAs and collected at the time of H2O2 treatment. (D) Densitometry analysis for p21 expression in three independent experiments corresponding to the results shown in Figure 2E. (E) Quantitation of the effect of DBC1, SIRT1, and HDAC3 siRNA on γ-H2.AX foci in 3T3-L1 preadipocytes after incubation with H2O2 (200 μm) shown in Figure 2F. Connecting lines show significant differences between conditions (P < 0.05, ANOVA, n = 3). (F) Chromatin immunoprecipitation (ChIP) for the p21 and p16 promoter regions in 3T3-L1 preadipocytes using an antibody against HDAC3. Nonspecific IgG was used as control. The results shown are the average ± SEM of 4 independent ChIP. (*P < 0.01; t-test). Data. S1 Methods. [file acel0013-0951-sd1.docx]

**Methods**

**Reagents and antibodies**. Unless otherwise specified, all reagents and chemicals were purchased from Sigma-Aldrich. Antibodies purchased were: SIRT1, Ac-H3(K9), (Cell signaling), HDAC3 (Abcam), DBC1 (Bethyl Laboratories), p53 and p21 (Santa Cruz), and Actin (Sigma).

**Animal handling and experiments*.*** All mice used in this study were maintained in the Mayo Clinic Animal Breeding facility. All experimental protocols were approved by the Institutional Animal Care and Use Committee at Mayo Clinic and all studies were performed according to the methods approved in the protocol. Female mice were fed with normal chow diet (diet no. 3807; KLIBA-NAFAG) from the moment of birth until 3 months of age, when they were placed on High-Fat Diet (AIN-93G, modified to provide 60% of calories from fat; Dyets Inc.), ad libitum and monitored for 12 weeks. Animals were kept in individual cages for the entire study. Body weight was recorded weekly and food intake was measured for 7 consecutive days with no difference detected between groups. In all experiments, groups consisted of at least 4 mice. In all experiments, only littermates were used. After 12 weeks of high-fat diet feeding, mice were euthanized and inguinal fat tissue was dissected under sterile conditions for primary cell culture, SA-βGal staining and mRNA isolation.

**Preadipocyte culture and differentiation.**

After 12 weeks fed with high-fat diet, female mice were euthanized with CO_2_ and inguinal fat depots were removed under sterile conditions. Fat tissue was minced into fragments, digested in Hanks' balanced salt solution with 1 mg/ml of type II collagenase (Worthington) for 60 min at 37°C, and filtered through a 100-μm nylon mesh. After digestion, mature adipocytes were separated from stromal vascular cells by centrifugation at 1000 × *g* for 10 min. After centrifugation the pellets were resuspended in α -MEM containing 10% Calf Serum (CFS) and antibiotics, and plated at density ∼4 × 104 cells/cm^2^. Cells were placed in humidified incubator with 3% oxygen. After 16 h, a period before replication occurs, adherent preadipocytes were washed, trypsinized, and replated at a density of 5 × 104 cells/cm^2^. Cells were cultured for 5 more days before harvesting for mRNA isolation or fixation for immunofluorescence.

**SA-βGal staining in tissue-** 50-100 mg of adipose tissue (AT) was rinsed in PBS immediately after biopsy and fixed for 10min in PBS containing 2% formaldehyde and 0.25% glutaraldehyde. After fixation, tissue was washed for 5 min two times in PBS and incubated for 18 hrs at 37^0^C in SA-βGal staining solution (1 mg/ml of 5-bromo-4-chloro-3-indolyl galactopyranoside, 0.12 mmol/l potassium ferrocyanide, 0.12 mmol/l potassium ferricyanide, 150 mM sodium chloride, and 1 mmol/l MgCl_2_ [pH 6]). The enzymatic reaction was stopped with cold PBS. After washing, AT pieces were incubated for 10 min with 10 μg/ml Hoescht 33342 to stain nuclei. They were placed between two mounting slides and images were taken from 8-10 random fields using phase contrast and fluorescence settings with a Nikon Eclipse Ti system. SA-β gal positive cells were counted in each field and numbers were normalized to total number of nuclei. Independent personnel who were not aware of experimental treatments performed quantification of senescent cells in different fields.

**Cellular senescence in 3T3-L1 preadipocytes.** Cell cycle arrest and cellular senescence (measured by SA-βGal staining) in 3T3-L1 pre-adipocytes were stimulated by treatment with H2O2. Briefly, cells were plated at low density (400,000 cells in 60 mm plate). The next day, cells were incubated in basal DMEM media (no pyruvate) with H2O2 (200-400 μM) for 2 hours. After treatment, the medium was replaced with DMEM + 10% FBS and cells were maintained for 7-10 days before fixing and staining for SA-βGal activity. Transfection with siRNA in 3T3-L1 was performed as described before (Escande *et al*, J Clin Invest. 2010 Feb;120 (2):545-58). When treating in combination with siRNA, H_2_O_2_ (200 μM) exposure was performed 48 hours after initial siRNA transfection. For SA-βGal staining, cells were fixed for 10 minutes in PBS containing 2% formaldehyde and 0.25% glutaraldehyde. After fixation, dishes were washed for 5 min two times in PBS and incubated for 18 hrs at 37^0^C in SA-βGal staining solution (1 mg/ml of 5-bromo-4-chloro-3-indolyl galactopyranoside, 0.12 mmol/l potassium ferrocyanide, 0.12 mmol/l potassium ferricyanide, 150 mM sodium chloride, and 1 mmol/l MgCl_2_ (pH 6). The enzymatic reaction was stopped with cold PBS. SA βGal-positive cells were quantified blindly in 500 cells in each experiment. Each experiment was repeated at least 3 times.

**DNA damage staining**

Preadipocytes were isolated from WT and DBC1 KO females after 12 weeks of high-fat diet feeding. Cells were cultures for 5 days after isolation and later fixed and processed for immunofluorescence by standard techniques. For DNA damage quantitation, ten different fields were counted per experimental condition or mouse (>400 cells). Cells with more than 4 foci were considered positive

**Chromatin Immunoprecipitation (ChIP) Assay**

ChIP was completed as previously described (Seo *et al* JBC 2012, vol 287, no 16, p12723-12735).  Briefly, 1X10^6^ cells were treated with formaldehyde to cross-link chromatin and then lysed in buffer containing protease inhibitors.  Nuclear extracts were sonicated and immunoprecipitated with appropriate antibodies: HDAC3 (Abcam), DBC1 (Bethyl) or IgG (SIGMA).  Chromatin complexes were collected with Protein A magnetic beads (Diagenode) and eluted (1% SDS, 0.1M NaHCO3).  After reverse cross-linking and purification, DNA samples were tested by real time PCR with SYBR Green (SAbiosciences) for enrichment of proteins in the promoter regions of the target genes, p16 and p21.  Primers were designed in the -3 kb to -1kb region upstream of the transcription start site of each gene.  Primers sequences are as follows:  p21 -3kb sense, 5’-GGGCAGTTTTGACATCCTGT-3’; p21 -3kb antisense, 5’-GACAGGCCTCCTCTTCTGTG-3’;p21 -2.5kb sense, 5’-ACAGCTTCTCCAAAGCAGGA-3’; p21 -2.5kb antisense, 5’-CATCTGTAATCCCGGCACTC-3’; p16 -1.8kb sense, 5’-TGCGGTTACTTCTCATCCAA-3’;

p16 -1.8kb antisense, 5’-CTGCTGCTCCTCCAGGTATC-3’; p16 -1.2kb sense, 5’-GCCTTTGCTGGGTTAGTTTG-3’; p16 -1.2kb antisense, 5’-CTGAGACTTGGTGGCCTTTC-3’; p16 -1kb sense, 5’-TTTCTCCTTCCCCCACTTTT-3’; p16 -1kb antisense, 5’-AGCCCGAGGACGTTGTTTAT-3’.  ChIP was completed four times with all samples normalized to input and HDAC3 or DBC1 graphed as fold enrichment compared to the IgG control sample.  * indicates p-values <0.01.

**Statistics.** Values are presented as mean ± SEM of 3–5 experiments, unless otherwise indicated. The significance of differences between means was assessed by ANOVA or 2-tailed Student’s t test, as indicated. A P value less than 0.05 was considered significant.

Supplementary figure 1


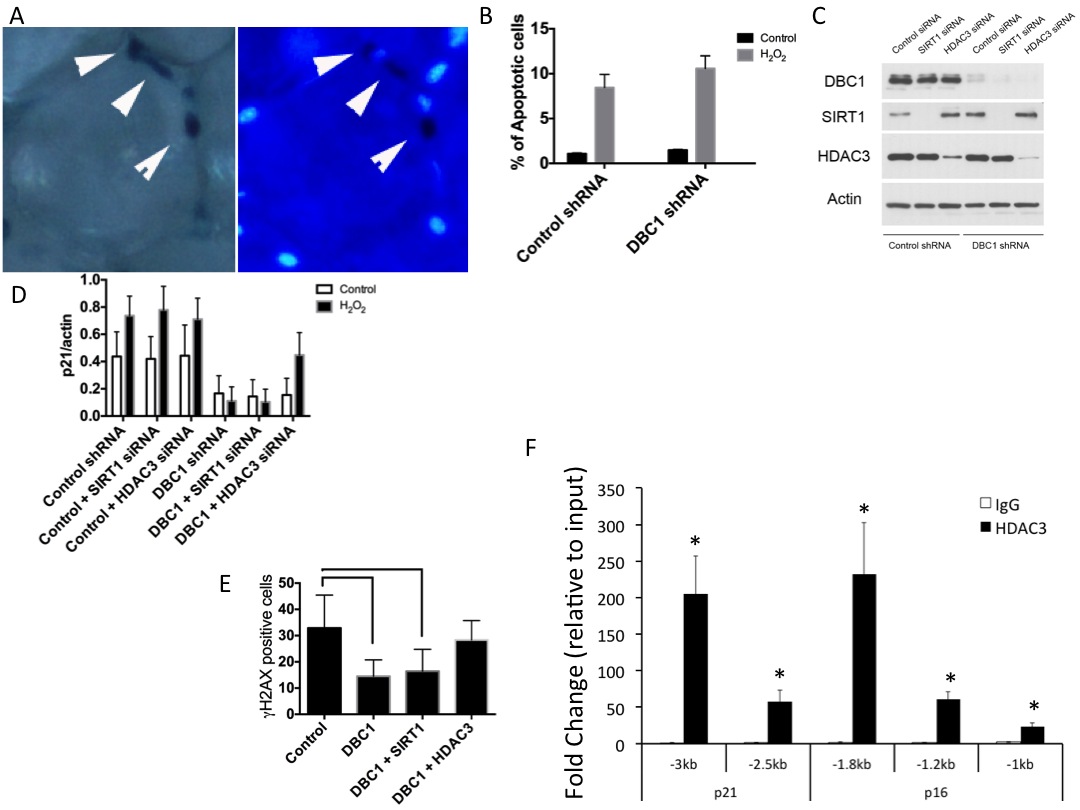


Supplementary figure 1 - A) DAPI counterstaining of fat tissue SA-βGal staining described in figure 1D, showing cytoplasmic localization of the βGal signal. B) Effect of DBC1 knockdown on apoptosis triggered by H_2_O_2_ in 3t3-L1 pre-adipocytes. Cells were incubated with 200 μM H_2_O_2_ for 2 hours, washed and let them recover for 4 more hours. Apoptosis was determined by nuclear shape using DAPI as nuclear marker. Pictures were taken blindly before and after treatment and apoptosis was independently evaluated by counting cells in the field based in nuclear shape, size, and DNA condensation. Results shown represent average ± SEM of 3 independent experiments. C) Western blot for DBC1, HDAC3, and SIRT1 in H_2_O_2_–treated 3T3-L1 preadipocytes transfected with the different siRNAs and collected at the time of H_2_O_2_ treatment. D) Densitometry analysis for p21 expression in three independent experiments corresponding to the results shown in figure 2E. E) Quantitation of the effect of DBC1, SIRT1, and HDAC3 siRNA on γ-H2.AX foci in 3T3-L1 preadipocytes after incubation with H_2_O_2_ (200 μM) shown in figure 2F. Connecting lines show significant differences between conditions (p<0.05, ANOVA, n=3). F) Chromatin immunoprecipitation (ChIP) for the p21 and p16 promoter regions in 3T3-L1 preadipocytes using an antibody against HDAC3. Non-specific IgG was used as control. The results shown are the average ± SEM of 4 independent ChIP. (*p<0.01; t-test)
